# Flow-Through PolyHIPE Silver-Based Catalytic Reactor

**DOI:** 10.3390/polym13060880

**Published:** 2021-03-12

**Authors:** Rok Mravljak, Ožbej Bizjak, Benjamin Božič, Matejka Podlogar, Aleš Podgornik

**Affiliations:** 1 Department of Chemical Engineering and Technical Safety, Faculty for Chemistry and Chemical Technology, University of Ljubljana, SI-1000 Ljubljana, Slovenia; rok.mravljak@fkkt.uni-lj.si (R.M.); ob5661@student.uni-lj.si (O.B.); bb8249@student.uni-lj.si (B.B.); matejka.podlogar@ijs.si (M.P.); 2 Department for Nanostructured Materials, Jožef Stefan Institute, SI-1000 Ljubljana, Slovenia; 3 COBIK, Tovarniška 26, 5270 Ajdovščina, Slovenia

**Keywords:** 4-nitrophenol reduction, AGET ATRP, heterogeneous catalysis, Tollens reagent, silver

## Abstract

Catalytic reactors performing continuously are an important step towards more efficient and controllable processes compared to the batch operation mode. For this purpose, homogenous high internal phase emulsion polymer materials with an immobilized silver catalyst were prepared and used as a continuous plug flow reactor. Porous material with epoxide groups was functionalized to bear aldehyde groups which were used to reduce silver ions using Tollens reagent. Investigation of various parameters revealed that the mass of deposited silver depends on the aldehyde concentration as well as the composition of Tollens reagent. Nanoparticles formed on the pore surface showed high crystallinity with a cuboctahedra crystal shape and highly uniform surface coverage. The example of the 4-nitrophenol catalytic reduction in a continuous process was studied and demonstrated to be dependent on the mass of deposited silver. Furthermore, productivity increased with the volumetric silver density and flow rate, and it was preserved during prolonged usage and storage.

## 1. Introduction

While most experiments in heterogeneous catalysis involving high internal phase emulsion polymer (polyHIPE) materials were performed in batch mode [1,2,3,4,5], flow reactors are currently gaining interest due to certain advantages such as increased mass and heat transfer and easy scale-up [6]. Some research groups have already demonstrated the efficient application of polyHIPEs in flow-through heterogeneous catalysis [7], including enzymatic ones [8]. When metal acts as a catalyst, its preferred form are nanoparticles providing a high surface area. They can be introduced onto the polymer pore surface via immobilization [9,10]. However, their high tendency toward agglomeration can result in a decrease in their specific surface [11], non-uniform surface coverage, or even pore clogging. Furthermore, poor attachment causes leakage of the metal nanoparticles, affecting its catalysis activity and efficiency [2]. An alternative to immobilization is the formation of the catalyst nanoparticles directly on the pore surface, for example, by reducing the dissolved ions from the solution [5,12,13,14].

Silver nanoparticles (AgNPs) are an example of a catalyst, which are known for their performance in heterogenous catalytic organic transformations [15]. Depending on the size and shape of the nanoparticles, different catalytic activities have been observed [16,17]. The model reaction most often used to evaluate their catalytic performance is the reduction of 4-nitrophenol (4-NP) to 4-aminophenol (4-AP) with NaBH_4_ [17,18]. 4-NP is one of the most common pollutants in the fabrication of pesticides, herbicides, and dyes [19]. It is often found in industrial and agricultural effluents and is stable and fairly soluble in water [20]. The product 4-AP is an important industrial intermediate in the preparation of analgesic and antipyretic drugs, used in corrosion inhibition and photo development, and as a hair-drying agent [19,20,21].

To avoid immobilization challenges with nanoparticles on a porous polymer, we tried to form them directly on highly porous high internal phase emulsion polymers (polyHIPEs). PolyHIPE supports have already shown to be a promising material for immobilization, exhibiting high porosity, low density, and high flexibility in morphology [1,2,3,4,5,22,23]. Furthermore, due to its open structure, the predominant transport mechanism is convection, minimizing diffusional limitations to active sites [8,24,25]. Due to negligible mass transport resistance, they enable studies of catalytic reaction kinetics at constant or variable conditions, providing new insights into the reaction mechanism [26]. Attachment of silver nanoparticles on their pore surface can be achieved through various functional groups such as amines [5], aldehydes [27,28], or chelating agents [12] that can act as nucleation sites and anchors for nanoparticles formed via established wet chemistry techniques [29]. In this study, we focused on the formation of silver nanoparticles (AgNPs) on the pore surface of polyHIPE material intended for the continuous conversion of 4-NP in the flow-through process. The catalytic performance of the AgNPs was evaluated by means of productivity and catalyst stability.

## 2. Materials and Methods

### 2.1. Materials

Poly(ethylene glycol)-*block*-poly(propylene glycol)-*block*-poly(ethylene glycol) or Synperonic^®^ L 121 (Sigma-Aldrich, St. Louis, MO, USA) (PL121), glycidyl methacrylate (Sigma-Aldrich) (GMA), ethylene glycol dimethacrylate (Merck KGaA, Darmstadt, Germany) (EGDMA), calcium chloride dihydrate (Honeywell Fluka, Charlotte, NC, USA) (CaCl_2_ · 2H_2_O), ethanol (Kefo, Ljubljana, Slovenia) (EtOH), methanol (Carlo Erba Reagents, Barcelona, Spain) MeOH, 4-vinylbenzyl chloride (90%, Sigma-Aldrich) (VBC), N,N,N’,N’’,N’’-pentamethyl-diethylenetriamine (Merck KGaA) (PMDETA), copper (II) chloride dihydrate (Merck KGaA) (CuCl_2_ · 2H_2_O), L(+)-Ascorbic Acid (Merck KGaA), Copper (I) chloride (Honeywell Fluka) CuCl, phenyl bis(2,4,6-trimethyl benzoyl)phosphine oxide (BASF Schweiz AG, Kaisten, Switzerland) (IC819), acetone (Honeywell Fluka), sodium (meta)periodate (Sigma-Aldrich) NaIO4, sulfuric acid (Sigma-Aldrich) H_2_SO_4_, sodium hydroxide (Honeywell Fluka) NaOH, 37% hydrochloric acid (Honeywell Fluka) HCl, silver nitrate (Honeywell Fluka) AgNO_3_, 25% ammonia solution (Carl Roth, Karlsruhe, Germany) NH_3_, 4-nitrophenol (Acros Organics, Fair Lawn, NJ, USA) (4-NP), sodium borohydride (Nokia Chemicals) NaBH_4_, and Hydrogen peroxide (Carlo Erba Reagents) H_2_O_2_. All chemicals were analytical grade if not specified otherwise and used as received. Deionized water was filtered through a 0.22 µm filter before use.

### 2.2. PolyHIPE Preparation

**Scheme 1:**
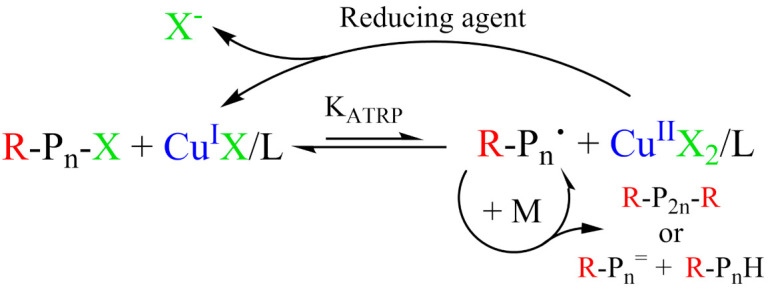
Reaction mechanism of AGET ATRP reaction. The Cu(II) in the halide and/or halide–ligand complex is reduced with the addition of a reducing agent to form Cu(I) which is the active compound reacting with the halogen on the initiator and later polymer chain. Stable radicals in equilibrium with the halogenated polymer chains are formed from the oxidation of Cu(I) to Cu(II) in the ligand complex. The cycle continues until all of the reducing agent and/or monomer is consumed. Further, the mechanism is the same as in conventional radical polymerization.

Activators generated by electron transfer (AGET) atom transfer radical polymerization (ATRP) is an alternative to the more commonly used free radical polymerization for the preparation of polyHIPE materials [30,31,32]. AGET ATRP shown in Scheme 1 was chosen to minimize the thermal destabilization of the high internal phase emulsion (HIPE) [33], promoting uniformity of the material.

A polymer with a nominal porosity of 0.80 was prepared. To make 100 mL of HIPE, we prepared the organic mixture containing 14.587 g GMA, 6.686 g EGDMA, 2.323 g PL121, 127 µL VBC, 14 µL PMDETA, and 0.106 g IC819. The composition of the aqueous phase was 77.467 g demi water, 0.775 g CaCl_2_ · 2H_2_O, and 38 µL of 0.3 g/mL aqueous solution of CuCl_2_ · 2H_2_O. After the entire aqueous phase was slowly added to the organic mixture during mixing with a paddle mixer at 400 rpm, 20 mL of the emulsion was poured into a 50 mL centrifuge tube and 100 µL of 0.04 g/mL AA/MeOH was added for every 10 mL of HIPE. The centrifuge tube was closed, shaken for 5 s, placed on a vortex mixer (vortex 3, IKA, Königswinter, Germany) at maximum rotation speed for 5 s to remove air bubbles, and poured into the polymerization housing. The housing was irradiated between two UV lights (light intensity ≈ 13 mW/cm^2^, wavelength maximum at 365 nm) for 5 min to trigger polymerization and left to polymerize at room temperature. The HIPE was observed to harden already after 10 min; however, the polymer was left for 3 days to facilitate high (complete) conversion with AGET ATRP. The obtained polymer was cut into 5–6-mm-long samples with a diameter of 11 mm (volume between 0.45 and 0.55 mL) and washed extensively with 20 vol.% EtOH before further use.

### 2.3. Homogeneity of the PolyHIPE

Hydrodynamic properties and homogeneity were evaluated with pulse experiments [34] and scanning electron microscopy (SEM) imaging. An amount of 40 μL of diluted aqueous acetone solution was injected at a flow rate of 1–3 mL/min on the high-performance liquid chromatography (HPLC) equipment (ÄKTAexplorer, Uppsala, Sweden). Homogeneity of the prepared polyHIPE material was checked using a field-emission scanning electron microscope (FEG-SEM, JEOL JSM 7600 F, Jeol Inc., Tokyo, Japan).

### 2.4. Functionalization

PolyHIPE functionalization was performed by conversion of epoxides from GMA to aldehydes. The polymer samples were immersed into 0.05–0.40 M NaIO_4_ containing 0–400 mM H_2_SO_4_ solution for 4 h at 60 °C [35,36]. After the reaction, samples were washed with filtered demi water and the wet mass of the polymer was measured.

### 2.5. Determination of Aldehyde Group

Three samples prepared with 0.4 and 400 mM H_2_SO_4_ were used to investigate the content of formed aldehydes groups. Titration was performed using 5 mM HCl and 5 mM NaOH solution filtered through a 0.20 μm filter. The samples were titrated in a flow-through mode on an HPLC system (Äkta Explorer, Uppsala, Sweden) at a flow rate of 3 mL/min. After titration, all three samples were immersed in 30 % H_2_O_2_ for 3 days at room temperature to oxidize aldehyde to carboxylic groups. H_2_O_2_ was selected due to its high reduction potential and absence of poorly soluble compound formation that might potentially contaminate the sample [37]. The titration was repeated after washing the H_2_O_2_-treated samples extensively with filtered demi water using the same reagents.

### 2.6. Formation of Silver Nanoparticles

AgNPs were prepared using Tollens reagent [38]. To produce the linear diamine silver (I) complex [Ag(NH_3_)_2_]^+^ in aqueous solution, 1.5 mL of 5 M NaOH was added to 3 mL of 1 M AgNO_3_. The formed precipitate (Ag_2_O) was then dissolved with the addition of 3 mL of 13.3 M NH_3_ followed by mixing on a vortex mixer if necessary (vortex 3, IKA, Germany) and adjusting to 10 mL (final concentration: 0.3 M AgNO_3_, 0.75 M NaOH, 4 M NH_3_). The ratio of NaOH and NH_3_ was altered to study the influence on nanoparticle growth. The concentration of AgNO_3_ and solution volume were kept constant for all experiments except for the AgNO_3_ concentration study and the time study of AgNPs growth where the composition was halved, or in samples prepared for the catalytic study where AgNO_3_ concentrations were higher. Samples were kept in solution for 1 day except for the time study of AgNPs growth.

After elapsed reaction time, the samples were put into 50 mL of 1 M NH_3_ for 3 days to stop the reaction and prevent precipitation of Ag_2_O. Finally, the samples were washed extensively with filtered demi water and the wet mass of the polyHIPE silver composite was measured.

The catalytic performance was tested for three samples with different amounts of AgNPs prepared at constant conditions. PolyHIPE samples were prepared with 4 mM H_2_SO_4_ and 0.1 M NaIO_4_ at various concentrations of AgNO_3_ (0.08, 0.16, and 0.32 M). The ratio of NH_3_ and NaOH was the same as above. The reaction time was 5 days, and the volume of the solution was 50 mL.

### 2.7. Catalytic Reduction of 4-NP

The reaction solution was prepared by dissolving 4-NP and NaBH_4_ in 0.1 M NaOH. The high pH of the solution was chosen to prevent the decomposition of NaBH_4_ and to convert 4-NP into the ionized form 4-nitrophenolate. The solution was filtered through a 0.20 μm filter and purged with N_2_ at a flow rate of 50 mL/s through a ceramic frit for 30 min to remove the dissolved oxygen and then degassed to remove the dissolved N_2_. Degassing was necessary to prevent disturbances in the UV–Vis cell due to the formation of N_2_ gas bubbles. To precisely determine absorption maximums of reagents and products, absorption spectra of a reaction solution containing 0.1 mM 4-NP and 15 mM NaBH_4_ in 0.1 M NaOH (pH = 13) pumped through the catalytic reactor with a syringe pump (PHD 4400, Harvard apparatus, Holliston, MA, USA) were measured after catalysis with a UV–Vis spectrophotometer (Tecan Infinite M200 Pro, Männedorf, Switzerland) with a light path of 1 cm. Values were used to set three online absorbance measurement wavelengths of the HPLC UV-detector (Äkta Explorer, Uppsala, Sweden) with a light path of 0.2 cm. The HPLC system was used to study the properties of the catalytic reactor with a reaction solution containing 0.4 mM 4-NP and 60 mM NaBH_4_.

After the reaction cycle was completed, the catalytic reactor was washed with 10–50 mL 0.1 mM 4-NP in 0.1 M NaOH until the UV–Vis response stabilized to remove adsorbed NaBH_4_. Removing NaBH_4_ was necessary to prevent the formation of a precipitate. Finally, samples were washed with 50 mL demi water and stored in 0.1 M NaOH.

### 2.8. Characterization of AgNPs

The deposited material was analyzed to identify the crystalline phase by X-ray diffraction analysis (XRD, X′Pert PRO high-resolution X-ray diffractometer; PANalytical B.V., Almelo, The Netherlands) with an Alpha1 configuration in the 2θ range 30°–90°, with a step of 0.034°/100 s using a fully opened X′Celerator detector and Cu-Kα radiation (1.5406 Å). The measured XRD pattern was compared to diffractograms from the JCPDS database (ICDD; International Centre for Diffraction Data, Newtown Square, PA, USA). The size and morphology of AgNPs were analyzed using field-emission scanning electron microscopes (FEG-SEM, JEOL JSM 7600 F, Jeol Inc., Tokyo, Japan and FE-SEM, Zeiss ULTRA plus, ZEISS, Oberkochen, Germany). SEM images were analyzed with ImageJ software to determine the AgNP and polyHIPE pore size distributions. Gravimetric analysis was performed to determine the mass of deposited AgNPs on the polyHIPE material. Samples were washed with filtered demi water and excess water was wiped off with a paper towel before weighing. The mass of the wet polyHIPE was subtracted from the mass of the wet polyHIPE containing AgNPs to obtain the mass of immobilized AgNPs. Three replicates of each experiment were prepared to calculate the average mass of the formed catalyst.

## 3. Results and Discussion

### 3.1. Homogeneity of the PolyHIPE

**Figure 1:**
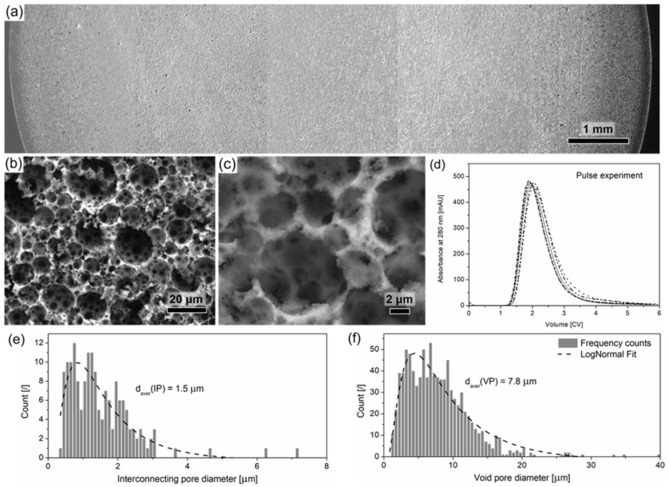
SEM images of the polyHIPE cross-section (**a**) and its microstructure at higher magnifications (**b**,**c**). Corresponding pulse experiment results performed at different flow rates are shown in (**d**). Bottom are presented pore size distributions, frequency counts, and average pore size for interconnecting (**e**) and void (**f**) pores.

PolyHIPE polymers exhibited a typical microstructure and high uniformity. In Figure 1a, porous structure uniformity is demonstrated via five assembled SEM images showing the cross-section of the entire polymer (at 35× magnification) and further confirmed by uniform peaks shown in Figure 1d obtained by the overlapping pulse response experiments performed at different flow rates [34]. The higher-magnification SEM images in Figure 1b,c show the open polyHIPE morphology with spherical void pores (VPs) connected with circular interconnecting pores (IPs) with the corresponding pore size distributions fitted with the log-normal distribution curve (Figure 1e,f). The absence of dead-end pores proves that the entire surface can be fully accessed by convection [34], and therefore no diffusion limitations, which would potentially affect the catalytic reaction, are expected.

### 3.2. Growth of Silver Nanoparticles

The interplay of the reaction rate and diffusion was found to affect the structure of silver nanoparticles, forming polyhedrons, dendrites, and almost amorphous crystals [39,40,41]. Therefore, reaction parameters were investigated to observe their effect on the quantity and morphology of AgNPs. To avoid any pore blockage and provide high deposition uniformity, AgNP formation should be initiated only on the pore surface; therefore, proper reaction conditions are of the utmost importance. While there are several routes to produce AgNPs [42,43,44], only a few report their formation on the surface, avoiding simultaneous formation in the bulk solution [12,45]. The formation of AgNPs is based on silver ion reduction; therefore, the surface has to bear groups capable of being easily oxidized. Due to the inherent presence of epoxy groups from a glycidyl methacrylate used in polyHIPE preparation [46], one option seems to be aldehyde groups. Their presence is determined via Tollens reagent, through which elementary silver is formed [38], and has already been demonstrated to deposit silver on different surfaces [27,28]. We investigated if, via this reaction mechanism, AgNPs attached strongly to the polyHIPE surface can be obtained. The synthesis of Tollens reagent can be written as follows [38]:

(I) 2 AgNO_3_ + 2 NaOH → Ag_2_O(s) + 2 NaNO_3_ + H_2_O

(II) 2 AgNO_3_ + 2 NH_3_ + H_2_O → Ag_2_O(s) + 2 NH_4_NO_3_

Ag_2_O, formed under alkali conditions as an intermediate product [47], is slightly soluble, releasing Ag^+^ which forms the complex with NH_3_ until most of the oxide is dissolved:

(III) Ag^+^ + 2 NH_3_ ⇌ [Ag(NH_3_)_2_]^+^

The formed [Ag(NH_3_)_2_]^+^ can react with aldehyde groups present on the polyHIPE surface, resulting in AgNP nucleation:

(IV) R-CHO + 2 [Ag(NH_3_)_2_]^+^ + 3 OH^-^ → R-COO^-^ + 2 Ag^0^ + 4 NH_3_ + 2 H_2_O

Nuclei are formed from the reduction of the [Ag(NH_3_)_2_]^+^ complex according to Reaction IV because the reduction potential of surface-bound aldehyde, E^0^ ≈ −0.2 V (vs. standard hydrogen electrode (SHE)) [48], is lower compared to E^0^([Ag(NH_3_)_2_]^+^) = +0.373 V (vs. SHE) [49]. Formation of AgNPs on the polyHIPE pore surface according to the above mechanism requires the introduction of aldehyde groups on polyHIPE as well as reaction conditions avoiding the presence of any insoluble Ag_2_O nanoparticles. There are several possible schemes to introduce aldehyde groups on methacrylate polymer pore surfaces [50]. The most common one is the hydrolysis of epoxy groups into vicinal hydroxyl groups followed by their oxidation. This is achieved through ring opening of epoxy groups using acids [51], especially H_2_SO_4_ [35], followed by oxidation using NaIO_4_ [52]. 

**Figure 2:**
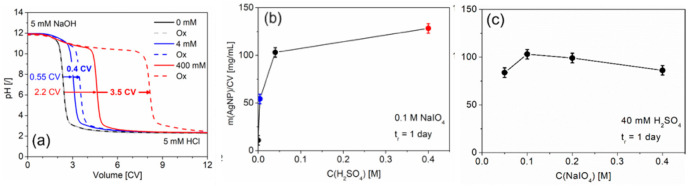
(**a**) Titration curves before and after the oxidation with H_2_O_2_. Arrows show the change in column volume (CV, volume divided by the volume of the column) in titration length between both reaction steps. (**b**) Normalized mass of deposited AgNPs vs. H_2_SO_4_ concentration. Blue and red dots show samples that were used for the titration. (**c**) Normalized mass of deposited AgNPs vs. NaIO_4_ concentration. Mass of AgNPs is normalized to CV (≈0.5 mL). The maximal theoretical m(AgNP)/CV was ≈690 mg/mL and the reaction time was 1 day.

Therefore, the influence of the H_2_SO_4_ and NaIO_4_ concentration effect on aldehyde group formation on the polyHIPE polymer was investigated. The effect of different H_2_SO_4_ concentrations in 0.1 M NaIO_4_ solution was explored, a concentration commonly used for the preparation of aldehyde groups intended for protein immobilization [52]. The amount of ionizable groups was determined by a flow-through titration, as described in Section 2.5. Results are presented in Figure 2a. Solid lines represent titration of the polymer after H_2_SO_4_ treatment only. One can see a shift in the titration curve with an increase in the H_2_SO_4_ concentration as a consequence of ester bond cleavage forming COOH groups. To determine the content of aldehyde groups obtained by simultaneous treatment of the polymer with H_2_SO_4_ and NaIO_4_, they were oxidized by using H_2_O_2_ to form carboxyl groups (see Section 2.5), which were, together with COOH groups formed by ester bond cleavage, again determined by titration (dashed lines in Figure 2a). From the overlapping of the solid and dashed lines, obtained for the polyHIPE sample treated with the absence of H_2_SO_4_, it can be concluded that no aldehyde groups were formed, demonstrating that epoxy group ring opening is essential for their formation. On the other hand, even at low H_2_SO_4_ concentrations, a significant amount of aldehyde groups were formed (the difference between dashed and solid lines), further increasing with higher concentrations. Furthermore, the ratio of aldehyde and COOH groups formed through ester bond cleavage increased (0.72 vs. 1.59 for 4 mM and 400 mM H_2_SO_4_, respectively).

Assuming that each aldehyde group has been oxidized into a COOH group via H_2_O_2_, their amount can be estimated to be 2.0 and 17.5 μmol/mL, respectively. If every aldehyde group would contribute to silver deposition through its reduction, the maximal mass of deposited AgNPs, based on electron stoichiometry (Reaction IV), would be 0.43 and 3.8 mg/mL for 4 mM and 400 mM H_2_SO_4_, respectively. Furthermore, if every aldehyde group acts as a nucleus for AgNP formation, their estimated average distance, taking into account the polyHIPE specific surface being in the range 3–20 m^2^/g [53] (0.72–5.2 m^2^/mL for polyHIPE density = 0.26 g/mL), was estimated to be between 0.3 and 2.2 nm. This simple calculation indicates that the nucleation center density would be extremely high. However, since it is not clear how many aldehyde groups represent nucleation sites, it is also not possible to predict the optimal aldehyde concentration. Subsequently, we investigated the effect of H_2_SO_4_ and NaIO_4_ concentrations on AgNP formation.

AgNPs were produced using a AgNO_3_, NaOH, and NH_3_ reagent solution described in Section 2.6. Initially, the deposition was investigated on polymer samples prepared by varying the H_2_SO_4_ concentration, keeping NaIO_4_ constant (Figure 2b). By an increase in H_2_SO_4_ concentration, the mass of deposited silver increased, confirming the importance of aldehyde groups in AgNP deposition. As expected, there was almost no silver present on the polyHIPE without aldehyde groups, while there was a steep increase for 0.04 M _2_SO_4_ and only a minor rise for the highest H_2_SO_4_ concentration. Due to that, experiments of varying NaIO_4_ concentrations were performed with 0.04 M H_2_SO_4_ (Figure 2c).

Results show almost no effect of the NaIO_4_ concentration on the quantity of deposited AgNPs, suggesting that already the lowest concentration provides sufficient aldehyde groups to cover the entire surface, a conclusion following the average aldehyde group distance estimation. Therefore, we decided to keep 0.1 M NaIO_4_ for all further experiments. On the other hand, H_2_SO_4_ was set to 4 mM, conditions where a limited amount of AgNPs were deposited, to enhance the effect of other reagents on AgNP formation.

**Figure 3:**
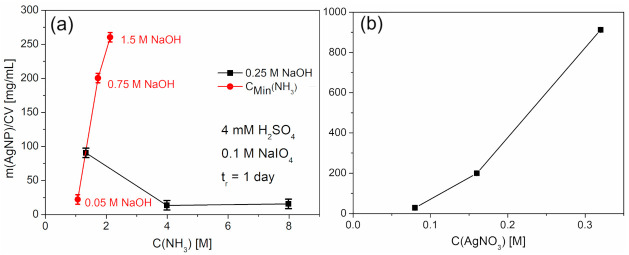
(**a**) Influence of NH_3_ and NaOH concentrations on the quantity of AgNPs. Black squares represent conditions with a constant NaOH concentration and red dots represent conditions with a minimal concentration of NH_3_ at various NaOH concentrations (written next to the data points) needed to dissolve the Ag_2_O precipitate. The maximal theoretical m(AgNP)/CV was ≈690 mg/mL and the reaction time was 1 day. (**b**) Influence of AgNO_3_ concentration on the quantity of AgNPs after 5 days of reaction time.

The deposition of AgNPs was further optimized by investigating the effect of NaOH and NH_3_ concentrations. In Figure 3a, two sets of experiments are shown. The black squares represent conditions where the NaOH concentration was constant and the NH_3_ concentration increased, while the red dots indicate a set where the NaOH concentration changed and the amount of NH_3_ added was just enough to dissolve the Ag_2_O precipitate completely. From the results, it can be concluded that a simultaneous increase in NH_3_ and NaOH concentrations increases the quantity of the deposited silver. On the other hand, an increase in only the NH_3_ concentration inhibited deposition and can therefore be used to quench the process, while an increase in only NaOH produced a Ag_2_O precipitate (data not shown).

Lastly, the impact of the AgNO_3_ concentration was tested. The NaOH/NH_3_ ratio was kept constant at 0.19 (first black square in Figure 3a) to be in the sensitive region of deposition. Results presented in Figure 3b show that, expectedly, AgNO_3_ has a significant impact on and caused a substantial increase in the deposited silver mass with its concentration.

For the highest NH_3_ and NaOH concentrations, the amount of deposited AgNPs was above 250 mg/mL (Figure 3a), while for the highest AgNO_3_ concentration, this value was even above 900 mg/mL (Figure 3b). These values are over two orders of magnitude higher than the maximal amount estimated based on the electron balance from the oxidation of aldehyde groups. Clearly, aldehyde oxidation can only act as a nucleation center while further AgNPs growth should proceed via other mechanisms of silver reduction, probably through water (hydroxide ion) oxidation [54], in the presence of silver nuclei [55]:

(V) 4 Ag(NH_3_)_2_^+^ + 4 OH^-^ ⇌ 4 Ag^0^ + O_2_(g) + 8 NH_3_ + 2 H_2_O

The occurrence of this silver reduction mechanism was supported by the formation of gas bubbles formed during the silver deposition for long reaction times. The higher reduction potential of water oxidation comparing to aldehyde groups [51] and the required presence of silver nuclei [55] also explain the preferential nucleation of silver on the polyHIPE surface.

**Figure 4:**
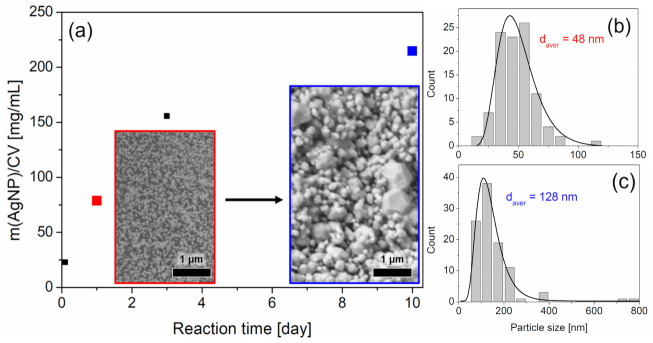
(**a**) Time-dependent growth of AgNP crystals on the surface. SEM images with the same scale for samples represented by the red and blue squares are shown for AgNP size comparison. (**b**,**c**) Corresponding size distributions determined from SEM images. The average diameter was determined from the fitted log-normal distribution curve. The maximal theoretical m(AgNP)/CV was ≈350 mg/mL. Samples were prepared with 4 mM H_2_SO_4_.

The time-dependent formation of AgNPs was studied by allowing the deposition process to proceed for several days. In Figure 4a, data obtained from four identical polymer samples containing 2 μmol/mL aldehyde groups, treated by the same reaction mixture, are shown. An expected increase in the deposited silver mass can be seen, while SEM images also reveal an increase in AgNP size and broadening of the size distribution (Figure 4b,c), probably due to Ostwald ripening [43]. Furthermore, for long reaction times, AgNPs exhibited distinct crystal faces, revealing that the AgNPs are probably single-crystalline.

**Figure 5:**
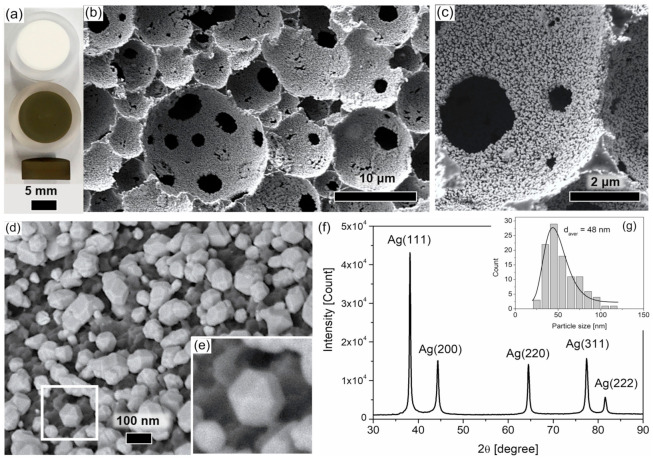
(**a**) Photograph of prepared polyHIPE with AgNPs. (**b**–**d**) SEM images showing AgNP crystals grown from the polyHIPE pore surface at four magnifications. A cuboctahedron-shaped AgNP is magnified (**e**) showing its attachment to polymer ridges on the pore surface. XRD pattern of the AgNP crystals. (**f**) shows high crystallinity of the composite polyHIPE and all characteristic refraction peaks for silver. (**g**) AgNP particle size distribution with calculated average diameter from the fitted log-normal distribution curve. The sample was prepared with 4 mM H_2_SO_4_ (see Section 2.6). The reaction time was 1 day.

Importantly, for all tested conditions, silver was deposited uniformly over the entire polymer volume (Figure 5a–d). SEM pictures revealed that this is also true on a microscale. A closer look demonstrates that the surface of the polyHIPE pores is not smooth but exhibits multiple dents with ridges in between (darker surface between lighter silver crystals). Since the ridges extend into the pores, they represent the preferred diffusion positions for crystal growth. As a consequence, most of the silver crystals were formed there, allowing the formation of idiomorphic crystals shown in Figure 5d,e. Nucleation on the ridge tips (Figure 5e) results in crystals partially embedding the polymer surface, providing strong adhesion and long-term stability. The XRD diffractogram shown in Figure 5f clearly shows the presence of distinctive peaks typical for crystalline Ag at 2Theta 38.15° (111), 44.33° (200), 64.50° (220), 77.44° (311), and 81.58° (222), proving that AgNPs are, in fact, nanocrystals, exhibiting a cuboctahedron morphology. The size distribution shown in Figure 5g was found to be very similar to that shown in Figure 4b demonstrating high reproducibility of the prepared composite polyHIPE material.

### 3.3. Catalytic Performance

**Figure 6:**
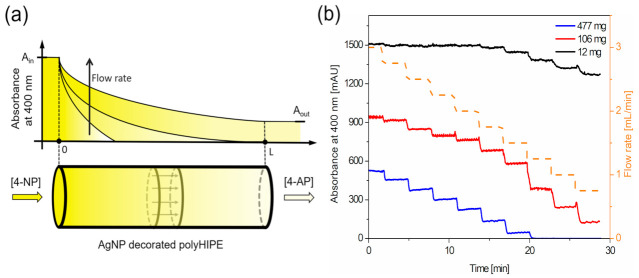
(**a**) Column schematic resembling continuous tubular reactor and absorbance profiles due to catalyzed reaction. (**b**) Experimentally measured absorbance at 400 nm (bold lines) for the reduction of 4-NP at flow rates between 0.75 and 3 mL/min (dashed line) is plotted. Absorbance corresponding to the inlet reagent solution was 1620 mAU. The baseline measured for a reaction solution without 4-NP was subtracted for all values. More details in Section 2.7.

To investigate the catalytic activity of the deposited nanocrystals, polymer samples treated with different AgNO_3_ concentrations, exhibiting the broadest range of deposited silver, were studied (see Figure 3b). The efficiency of the silver catalyst was tested by reduction of 4-NP to 4-AP, which is an extensively investigated process commonly used for catalyst activity tests [17,18,19,56,57,58,59,60]. The reaction was monitored by in-line measurement of absorbance at the column outlet (Figure 6a). A decrease in absorbance at 400 nm (4-NP absorption maximum) in comparison to the inlet reagent solution indicated the conversion of 4-NP. The reagent concentrations were chosen from preliminary experiments by varying NaBH_4_ (3–60 mM) and 4-NP (0.1–1 mM) concentrations (data not shown) to find the conditions at which, for all polymer samples, the reaction can be monitored accurately, despite significant differences in catalyst loading. The flow rate of the reagent through the catalyst defined the residence time for the reaction; therefore, absorbance at 400 nm expectedly decreased at lower flow rates (Figure 6b). In addition, the reaction proceeded faster for higher amounts of the AgNP catalyst.

The reaction mechanism of 4-NP conversion is rather complex. It depends on both reactants NaBH_4_ and 4-NP that compete for the reaction sites on the catalyst surface, resulting in optimal conditions where the reaction rate is at the maximum [57]. The reaction was described with the Langmuir–Hinshelwood mechanism [57,58]. However, the reaction can be relatively accurately described as an irreversible equimolar reaction between 4-NP and NaBH_4_ to 4-AP [61]:

(VI) $$4-\mathrm{NP}\;+{\;\mathrm{NaBH}}_{4}\;\overset{\;cat.\;\;AgNP}{\to }4-\mathrm{AP}$$

Therefore, reaction kinetics can be written as

(1) $$r_{4-\mathrm{NP}}=-\;k\;\left[4-\mathrm{NP}\right]\;\left[{\mathrm{NaBH}}_{4}\right]=-\;k_{cat}\;m_{cat}\;\left[4-\mathrm{NP}\right]\;\left[{\mathrm{NaBH}}_{4}\right]$$

where *r*_4-NP_ is the rate of reaction [mol_A_ L^−1^ min^−1^], *k* is the rate constant [L mol_B_^−1^ min^−1^], *k_cat_* is the catalytic rate constant [L mol_B_^−1^ min^−1^ g^−1^], *m_cat_* is the mass of catalyst, and [4-NP] and [NaBH_4_] are the concentrations of reactants.

As the NaBH_4_ concentration was in high excess [61], a solution with high pH was used [56], and oxygen was removed [59,60]; therefore, Equation (1) can be further simplified into pseudo-first-order reaction kinetics with an apparent rate constant [56]:

(2) $$r_{4-\mathrm{NP}}=-k^{\prime }\;\left[4-\mathrm{NP}\right]=-\;k_{cat}^{’}\;m_{cat}\;\left[4-\mathrm{NP}\right]$$

for a continuous tubular reactor (Figure 6), resulting in the following expression:

(3) $$\frac{A_{out}}{A_{in}}=\frac{C_{out}}{C_{in}}=e^{-k^{\prime }\;t_{r}}=\;e^{-k_{cat}^{’}\;m_{cat}\;\frac{V_{c}\;\varepsilon }{F}}$$

where *k’* is the apparent rate pseudo-first-order rate constant [min^−1^], *k_cat_’* is the apparent rate pseudo-first-order catalytic rate constant [min^−1^ g^−1^], *A_in_*/*A_out_* and *C_in_*/*C_out_* are the absorbance and concentration ratios of 4-NP at the inlet and outlet of the column with subtracted baseline (solution without 4-NP), *F* is the flow rate, *V_c_* is the column volume, *ε* is the column porosity, and *t_r_* is the residence time.

**Figure 7:**
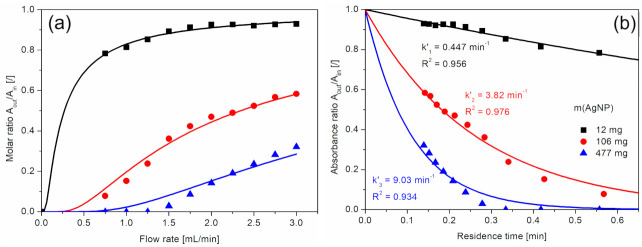
(**a**) The 4-NP absorbance ratio for in- and outflow vs. mobile phase flow rate. (**b**) Absorbance ratio vs. retention time for the determination of the apparent rate constant (*k’*) from regression analysis for samples with various loadings of catalyst (see Figure 3b) according to Equation (3).

As seen from Equation (3), the catalytic rate constant (*k’*) can be used as a measure of catalyst efficiency. In Figure 7a, the ratio *A_in_*/*A_out_* shows the molar ratio of the reactant at the column exit, and in Figure 7b, experimental data derived from Figure 6 are fitted by Equation (3) from where corresponding reaction constants *k’* are calculated. The *k’* values indicate that the reaction proceeds 8 times faster for the sample containing 106 mg AgNPs and 19 times faster for the sample containing 477 mg AgNPs comparing to the sample with 12 mg AgNPs. Reaction constants are hard to compare to literature data due to non-standardized reaction conditions [61]; nevertheless, they are listed in Table 1.

Reaction constants normalized to the catalyst mass show that the catalyst efficiency is similar for the samples with a low silver mass but only half for the sample with the highest deposited amount of AgNPs. This finding can be explained by considering that the reaction proceeds on the catalysis surface; therefore, its specific surface is important. From SEM images in Figure 4, it can be seen that for the highest loading, some crystals do agglomerate and some are very large, both contributing to a decrease in the catalyst specific surface area and, consequently, the reaction rate.

For evaluation of the catalysis applicative potential, it is reasonable to calculate its productivity when used as a tubular reactor operating in a continuous mode. Productivity (*Pr*) can be defined as

(4) $$\mathrm{Pr}=\frac{\left(C_{in}-C_{out}\right)\;F}{V_{c}}$$

Substituting *C_out_* from Equation (3) into Equation (4) gives

(5) $$Pr=C_{in}\left(1-e^{-k_{cat}^{’}\;m_{cat}\;\frac{\;V_{c}\;\varepsilon }{F}}\right)\;\frac{F}{V_{c}}$$

**Figure 8:**
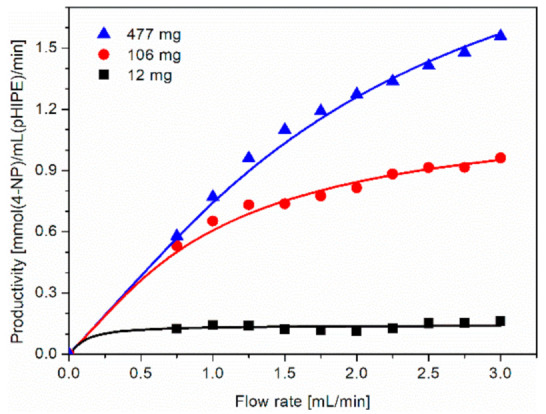
The productivity vs. mobile phase flow rate according to Equation (5). Reaction conditions and details are described in Section 2.7.

In Figure 8, the calculated productivity is shown. The amount of reactant leaving the reactor is inversely proportional to the flow rate due to longer retention times. Therefore, productivity increases. For the sample containing the lowest AgNPs mass, the plateau at which the productivity becomes constant is reached at much lower flow rates compared to the sample having a higher loading of AgNPs. Despite the less effective catalytic conversion based on *k_cat_* (Table 1), one can see that productivity is the highest for the highest silver mass, resulting in a more compact catalytic reactor.

**Figure 9:**
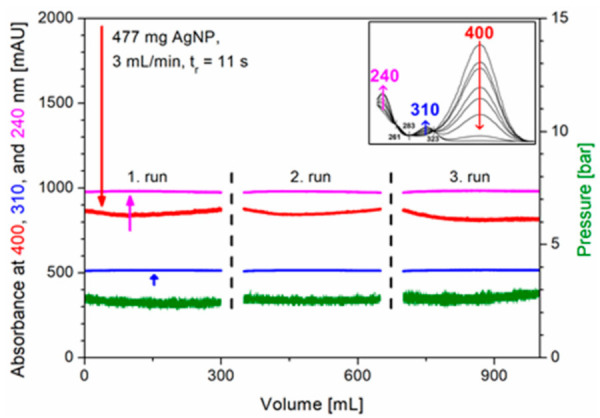
Stability of the polyHIPE with AgNPs in continuous mode showing three separate runs between storage in 0.1 M NaOH for 3 days. The graph in the upper right corner shows the characteristic absorbance peaks for the reaction. Reaction conditions and details are described in Section 2.7.

Needless to emphasize, continuous operation is only feasible when the catalytic performance is stable over a long time. Therefore, the stability test was performed on the sample with the highest productivity. Three repeated runs under the same reaction conditions lasting 100 min were performed. Results shown in Figure 9 indicate no changes in absorbance or pressure, even though the sample was reconditioned after every run and stored for 3 days in 0.1 M NaOH solution between runs. A pressure decrease would be expected if AgNPs wash off from the polymer pores, while an increase would occur if pores become clogged. However, neither was observed. Seemingly, the strong adhesion of AgNP crystals prevented catalyst leaking and a change in its catalytic activity over prolonged storage and re-usage.

## 4. Conclusions

Ideal catalytic reactors should possess, besides a selective catalyst, high porosity to allow high reactant volumetric utilization, high permeability to obtain a high throughput at a moderate pressure drop, a high surface area to provide high reaction kinetics, and absence of diffusion limitations to enable rapid transport to and from catalytic sites. Furthermore, to minimize the amount of catalysts, a matrix with proper characteristics for catalyst fixation is preferred. PolyHIPEs match these criteria due to their highly tunable morphology, open high porosity, and convection-based transport. In situ crystallization of a silver catalyst demonstrated the formation of exposed idiomorphic nanocrystals being strongly attached to the polymer pore surface, resulting in efficient long-term stable operation. Due to the high flexibility in tailoring a matrix as well as a crystal catalyst’s properties, their combination seems to be a powerful system for the preparation of various continuous catalytic reactors of high efficiency.

## Figures and Tables

**Table 1:** Reaction constants for the samples with various loadings of catalyst (see Figure 3b) calculated according to Equations (1) and (2). NaBH_4_ concentration was 60 mM.

m(AgNP) [g]	*k’* [min^−1^]	*k* [L mol_B_^−1^ min^−1^]	*k_cat_’* [min^−1^ g^−1^]	*k_cat_* [L mol_B_^−1^ min^−1^ g^−1^]
0.012	0.447	7.45	37.3	621
0.106	3.82	63.7	36.0	601
0.477	9.03	151	18.9	316

## References

[1] Liu H., Wan D., Du J., Jin M. (2015). Dendritic Amphiphile Mediated One-Pot Preparation of 3D Pt Nanoparticles-Decorated PolyHIPE as a Durable and Well-Recyclable Catalyst. ACS Appl. Mater. Interfaces.

[2] Ye Y., Jin M., Wan D. (2015). One-pot synthesis of porous monolith-supported gold nanoparticles as an effective recyclable catalyst. J. Mater. Chem. A.

[3] Zhu Y., Hua Y., Zhang S., Wang Y., Chen J. (2015). Open-cell macroporous bead: A novel polymeric support for heterogeneous photocatalytic reactions. J. Polym. Res..

[4] Lee J., Chang J.Y. (2020). A hierarchically porous catalytic monolith prepared from a Pickering high internal phase emulsion stabilized by microporous organic polymer particles. Chem. Eng. J..

[5] Yuan W., Chen X., Xu Y., Yan C., Liu Y., Lian W., Zhou Y., Li Z. (2018). Preparation and recyclable catalysis performance of functional macroporous polyHIPE immobilized with gold nanoparticles on its surface. RSC Adv..

[6] Plutschack M.B., Pieber B., Gilmore K., Seeberger P.H. (2017). The Hitchhiker’s Guide to Flow Chemistry. Chem. Rev..

[7] Ottens M., Leene G., Beenackers A.A.C.M., Cameron N., Sherrington D.C. (2000). PolyHipe: A New Polymeric Support for Heterogeneous Catalytic Reactions: Kinetics of Hydration of Cyclohexene in Two- and Three-Phase Systems over a Strongly Acidic Sulfonated PolyHipe. Ind. Eng. Chem. Res..

[8] Ruan G., Wu Z., Huang Y., Wei M., Su R., Du F. (2016). An easily regenerable enzyme reactor prepared from polymerized high internal phase emulsions. Biochem. Biophys. Res. Commun..

[9] Alshehri S.M., Almuqati T., Almuqati N., Al-Farraj E., Alhokbany N., Ahamad T. (2016). Chitosan based polymer matrix with silver nanoparticles decorated multiwalled carbon nanotubes for catalytic reduction of 4-nitrophenol. Carbohydr. Polym..

[10] Tan L., Tan B. (2020). Functionalized hierarchical porous polymeric monoliths as versatile platforms to support uniform and ultrafine metal nanoparticles for heterogeneous catalysis. Chem. Eng. J..

[11] Ding R., Chen Q., Luo Q., Zhou L., Wang Y., Zhang Y., Fan G. (2020). Salt template-assisted in situ construction of Ru nanoclusters and porous carbon: Excellent catalysts toward hydrogen evolution, ammonia-borane hydrolysis, and 4-nitrophenol reduction. Green Chem..

[12] Cheng W.M., Wang C.C., Chen C.Y. (2012). The influence of Ni nanoparticles and Ni(II) on the growth of Ag dendrites immobilized on the chelating copolymer membrane. Mater. Chem. Phys..

[13] Akay G., Calkan B. (2015). Preparation of Nanostructured Microporous Metal Foams through Flow Induced Electroless Deposition. J. Nanomater..

[14] Wan Y., Feng Y., Wan D., Jin M. (2016). Polyamino amphiphile mediated support of platinum nanoparticles on polyHIPE as an over 1500-time recyclable catalyst. RSC Adv..

[15] Dong X.Y., Gao Z.W., Yang K.F., Zhang W.Q., Xu L.W. (2015). Nanosilver as a new generation of silver catalysts in organic transformations for efficient synthesis of fine chemicals. Catal. Sci. Technol..

[16] Cao S., Tao F.F., Tang Y., Li Y., Yu J. (2016). Size- and shape-dependent catalytic performances of oxidation and reduction reactions on nanocatalysts. Chem. Soc. Rev..

[17] Liao G., Gong Y., Zhong L., Fang J., Zhang L., Xu Z., Gao H., Fang B. (2019). Unlocking the door to highly efficient Ag-based nanoparticles catalysts for NaBH_4_-assisted nitrophenol reduction. Nano Res..

[18] Kim J.G., Cha M.C., Lee J., Choi T., Chang J.Y. (2017). Preparation of a Sulfur-Functionalized Microporous Polymer Sponge and In Situ Growth of Silver Nanoparticles: A Compressible Monolithic Catalyst. ACS Appl. Mater. Interfaces.

[19] Yan Z., Fu L., Zuo X., Yang H. (2018). Green assembly of stable and uniform silver nanoparticles on 2D silica nanosheets for catalytic reduction of 4-nitrophenol. Appl. Catal. B Environ..

[20] Ma M., Yang Y., Li W., Feng R., Li Z., Lyu P., Ma Y. (2019). Gold nanoparticles supported by amino groups on the surface of magnetite microspheres for the catalytic reduction of 4-nitrophenol. J. Mater. Sci..

[21] Lv Z.S., Zhu X.Y., Meng H.-B., Feng J.J., Wang A.J. (2019). One-pot synthesis of highly branched Pt@Ag core-shell nanoparticles as a recyclable catalyst with dramatically boosting the catalytic performance for 4-nitrophenol reduction. J. Colloid Interface Sci..

[22] Zhang T., Sanguramath R.A., Israel S., Silverstein M.S. (2019). Emulsion Templating: Porous Polymers and Beyond. Macromolecules.

[23] Taylor-Pashow K.M.L., Pribyl J.G. (2019). PolyHIPEs for Separations and Chemical Transformations: A Review. Solvent Extr. Ion Exch..

[24] Thomas J.M., Thomas W.J. (2015). Principles and Practices of Heterogneous Catalysis.

[25] Rajamanickam V., Herwig C., Spadiut O. (2015). Monoliths in Bioprocess Technology. Chromatography.

[26] Trojanowicz M. (2020). Flow Chemistry in Contemporary Chemical Sciences: A Real Variety of Its Applications. Molecules.

[27] Montazer M., Alimohammadi F., Shamei A., Rahimi M.K. (2012). In situ synthesis of nano silver on cotton using Tollens’ reagent. Carbohydr. Polym..

[28] Textor T., Fouda M.M.G., Mahltig B. (2010). Deposition of durable thin silver layers onto polyamides employing a heterogeneous Tollens’ reaction. Appl. Surf. Sci..

[29] Diodati S., Dolcet P., Casarin M., Gross S. (2015). Pursuing the Crystallization of Mono- and Polymetallic Nanosized Crystalline Inorganic Compounds by Low-Temperature Wet-Chemistry and Colloidal Routes. Chem. Rev..

[30] Gurevitch I., Silverstein M.S. (2011). Nanoparticle-Based and Organic-Phase-Based AGET ATRP PolyHIPE Synthesis within Pickering HIPEs and Surfactant-Stabilized HIPEs. Macromolecules.

[31] Lamson M., Epshtein-Assor Y., Silverstein M.S., Matyjaszewski K. (2013). Synthesis of degradable polyHIPEs by AGET ATRP. Polymer.

[32] Simakova A., Averick S.E., Konkolewicz D., Matyjaszewski K. (2012). Aqueous ARGET ATRP. Macromolecules.

[33] Tadros T.F., Tadros T.F. (2013). Emulsion Formation and Stability.

[34] Mravljak R., Bizjak O., Podlogar M., Podgornik A. (2020). Effect of polyHIPE porosity on its hydrodynamic properties. Polym. Test..

[35] Mallik R., Jiang T., Hage D.S. (2004). High-Performance Affinity Monolith Chromatography: Development and Evaluation of Human Serum Albumin Columns. Anal. Chem..

[36] Telvekar V.N., Patel D.J., Mishra S.J. (2008). Oxidative Cleavage of Epoxides Using Aqueous Sodium Paraperiodate. Synth. Commun..

[37] Bockris J.O.M., Oldfield L.F. (1955). The oxidation-reduction reactions of hydrogen peroxide at inert metal electrodes and mercury cathodes. Trans. Faraday Soc..

[38] Tollens B. (1882). Ueber ammon-alkalische Silberlösung als Reagens auf Aldehyd. Ber. Dtsch. Chem. Ges..

[39] Yang T., Han Y. (2016). Quantitatively relating diffusion and reaction for shaping particles. Cryst. Growth Des..

[40] Yang T., Liu J., Dai J., Han Y. (2017). Shaping particles by chemical diffusion and reaction. CrystEngComm.

[41] You H., Ding C., Song X., Ding B., Fang J. (2011). In situ studies of different growth modes of silver crystals induced by the concentration field in an aqueous solution. CrystEngComm.

[42] Singh J., Mehta A., Rawat M., Basu S. (2018). Green synthesis of silver nanoparticles using sun dried tulsi leaves and its catalytic application for 4-Nitrophenol reduction. J. Environ. Chem. Eng..

[43] Thanh N.T.K., Maclean N., Mahiddine S. (2014). Mechanisms of Nucleation and Growth of Nanoparticles in Solution. Chem. Rev..

[44] Sadeghi S., Moghbeli M.R. (2012). Synthesis and dispersion of colloidal silver nanoparticles on microcellular polyHIPE support. Colloids Surfaces A Physicochem. Eng. Asp..

[45] Cao H.-L., Huang H.-B., Chen Z., Karadeniz B., Lü J., Cao R. (2017). Ultrafine Silver Nanoparticles Supported on a Conjugated Microporous Polymer as High-Performance Nanocatalysts for Nitrophenol Reduction. ACS Appl. Mater. Interfaces.

[46] Krajnc P., Leber N., Štefanec D., Kontrec S., Podgornik A. (2005). Preparation and characterisation of poly(high internal phase emulsion) methacrylate monoliths and their application as separation media. J. Chromatogr. A.

[47] Welham N.J., Kelsall G.H., Diaz M.A. (1993). Thermodynamics of Ag-Cl-H_2_O, Ag-Br-H_2_O and Ag-I-H_2_O systems at 298 K. J. Electroanal. Chem..

[48] Adkins H., Elofson R.M., Rossow A.G., Robinson C.C. (1949). The Oxidation Potentials of Aldehydes and Ketones. J. Am. Chem. Soc..

[49] Ho N.A.D., Babel S. (2019). Electrochemical reduction of different Ag(I)-containing solutions in bioelectrochemical systems for recovery of silver and simultaneous power generation. RSC Adv..

[50] Vlakh E.G., Tennikova T.B. (2013). Flow-through immobilized enzyme reactors based on monoliths: I. Preparation of heterogeneous biocatalysts. J. Sep. Sci..

[51] Wang C., Zhao S., Wei Y. (2012). Hydrophilic Modification of Microporous Polysulfone Membrane via Surface-Initiated Atom Transfer Radical Polymerization and Hydrolysis of Poly(glycidylmethacrylate). Chin. J. Chem..

[52] Křvenková J., Bilková Z., Foret F. (2005). Chararacterization of a monolithic immobilized trypsin microreactor with on-line coupling to ESI-MS. J. Sep. Sci..

[53] Hainey P., Huxham I.M., Rowatt B., Sherrington D.C., Tetley L. (1991). Synthesis and ultrastructural studies of styrene-divinylbenzene Polyhipe polymers. Macromolecules.

[54] Starovoytov O.N., Kim N.S., Han K.N. (2007). Dissolution behavior of silver in ammoniacal solutions using bromine, iodine and hydrogen-peroxide as oxidants. Hydrometallurgy.

[55] Henglein A. (1989). Non-metallic silver clusters in aqueous solution: Stabilization and chemical reactions. Chem. Phys. Lett..

[56] Grzeschik R., Schäfer D., Holtum T., Küpper S., Hoffmann A., Schlücker S. (2020). On the Overlooked Critical Role of the pH Value on the Kinetics of the 4-Nitrophenol NaBH_4_-Reduction Catalyzed by Noble-Metal Nanoparticles (Pt, Pd, and Au). J. Phys. Chem. C.

[57] Wunder S., Polzer F., Lu Y., Mei Y., Ballauff M. (2010). Kinetic Analysis of Catalytic Reduction of 4-Nitrophenol by Metallic Nanoparticles Immobilized in Spherical Polyelectrolyte Brushes. J. Phys. Chem. C.

[58] Menumerov E., Hughes R.A., Neretina S. (2016). Catalytic Reduction of 4-Nitrophenol: A Quantitative Assessment of the Role of Dissolved Oxygen in Determining the Induction Time. Nano Lett..

[59] Strachan J., Barnett C., Masters A.F., Maschmeyer T. (2020). 4-Nitrophenol Reduction: Probing the Putative Mechanism of the Model Reaction. ACS Catal..

[60] Neal R.D., Inoue Y., Hughes R.A., Neretina S. (2019). Catalytic Reduction of 4-Nitrophenol by Gold Catalysts: The Influence of Borohydride Concentration on the Induction Time. J. Phys. Chem. C.

[61] Iben Ayad A., Luart D., Ould Dris A., Guénin E. (2020). Kinetic Analysis of 4-Nitrophenol Reduction by “Water-Soluble” Palladium Nanoparticles. Nanomaterials.

